# A Systematic Study on Design Initiation of Conceptual 3DPVS

**DOI:** 10.3390/biomimetics4020031

**Published:** 2019-04-11

**Authors:** Haobo Yuan, Ke Xing

**Affiliations:** School of Engineering, University of South Australia; Mawson Lakes Blvd, Salisbury 5095, Australia; Ke.Xing@unisa.edu.au

**Keywords:** 3D scaffold, biomimetic scaffold, cell culture, 3DPVS, scaffold innovation, conceptual design, design initiation, GMBV characterization, base model, ideality of scaffold

## Abstract

An important product in biomedical and biomimetic engineering is the 3D scaffold, which mimics the real tissue in vitro to achieve the external cultivation of cells. The difference between the 3D scaffold and other biomimetic products lies in the fact that the former mimics the internal features of tissue, while the latter generally approximates the external traits of biological beings. In the field of scaffold engineering, the 3D printed vibratory scaffold, 3DPVS, has been proposed as a present-to-future novel scaffold product, and it currently stays at the stage of conceptual development. To achieve the novel design of the conceptual 3DPVS, a conceptual design process has been established by authors in their previous work, which contain three main stages, namely the design initiation, concept generation, and concept evaluation. In terms of design initiation, it is a ‘must-accomplish’ stage which generates outputs for both the subsequent concept generation and evaluation. Work of design initiation therefore is of significant value and it consists of several tasks; that is, conducting a thorough literature review, summarizing the fundamental issues preparing the general conceptual design, studying the multi-characterization of the 3DPVS, putting forward the potential base model(s), as well as indicating the ideality of the scaffold and establishing potential ideal model(s) for the 3DPVS. In this paper, design initiation will be chiefly focused upon these essential aspects to be discussed, work of which is expected to be useful in establishing a solid ground for future innovation work of the 3DPVS.

## 1. Introduction

Four aspects will be introduced in this section, in order to provide a better understanding of the paper’s background and its possible indications.

### 1.1. Basic Knowledge of Biomimetic Cell Culture Scaffolds

Basic knowledge of scaffold engineering will be firstly introduced. A cell culture scaffold is defined as a class of artificially created biomimetic products used for culturing cells in vitro through mimicking some real tissue properties. Scaffold engineering, in this connection, has developed in two directions, as studied by literature review [1]. One is ‘from static into dynamic’, with proven effects that dynamic cultures have benefits over static ones. Several attempts were made to endow scaffold cell culturing with dynamic properties, for example, connecting the scaffold with mechanical means such as external vibrators or shakers. This aims to create a biomimetic atmosphere which approximates part of the dynamicity inside real tissue. In another direction, scaffolds were developed by focusing on dimensional development, ‘2D into 3D’, so to speak. 3D scaffolds have been utilized to help external culturing mimic real tissue 3D environments and produce better performance compared with traditional 2D cell culture. These scaffolds basically have a nature of being passive (also called ‘static’), therefore resulting in several inevitable limitations, as summarized in [1].

### 1.2. Evolutional Ladder of Scaffold Engineering and the Positionality of the 3DPVS 

In a previous study [2], a multilayer evolutional ladder of scaffold engineering was established, indicating the traditional 2D scaffold and 3D scaffold as the lower part of ladder and the vibratory scaffold as the present-to-future product which occupies a higher position in the ladder of scaffold engineering. In this connection, a novel concept, namely the 3D printed vibratory scaffold (3DPVS), has been put forward and justified via studying previous traditional scaffolds as well as by simultaneously penetrating the ‘laws of system evolution’ (LSE) into scaffold engineering. In brief, the concept of the 3DPVS indicates that a trinity of the separate elements of vibration, scaffolding, and 3D printing (3DP) would turn into a unified system with promising vibratory function and 3DP fabricability endowed by the scaffold itself. This would, to a large extent, mitigate the limitations existing in current mechanisms which rely on external vibrators or bioreactors. Furthermore, the 3DPVS combines novel 3D printing technology together with vibration, transforming the role of the scaffold passively receiving vibration into a product actively generating vibration. Vibration, as one core element inside the 3DPVS, has been indicated as one of the most useful types of dynamicity currently required by scaffold engineering, and 3D printing (3DP), another core element, has been indicated as the technology bridging the gap between traditional scaffolds and future novel vibratory scaffolds. Consequently, the 3DPVS would adequately possess the existing merits of traditional scaffolds along with its innovative provision of tailored vibratory functions required by definite cell culture.

### 1.3. Introducing the Three-Stage Conceptual Design Process of the 3DPVS

In a previous study [3], the conceptual design of the 3DPVS was divided into three main stages, namely the design initiation, concept generation, and concept evaluation. The main task of the concept generation phase is to innovatively create possible solutions which would realize all or most of the desired functions expected for the 3DPVS. Following this, the evaluation phase is to judge to what extent the generated conceptual 3DPVS scaffold satisfies these proposed functions or requirements as well as to provide feedback for improving the design in a circular process. However, to guarantee the quality of these two stages, one stage must be completed at a relatively ideal level, and this stage is defined as design initiation. 

**Figure 1:**
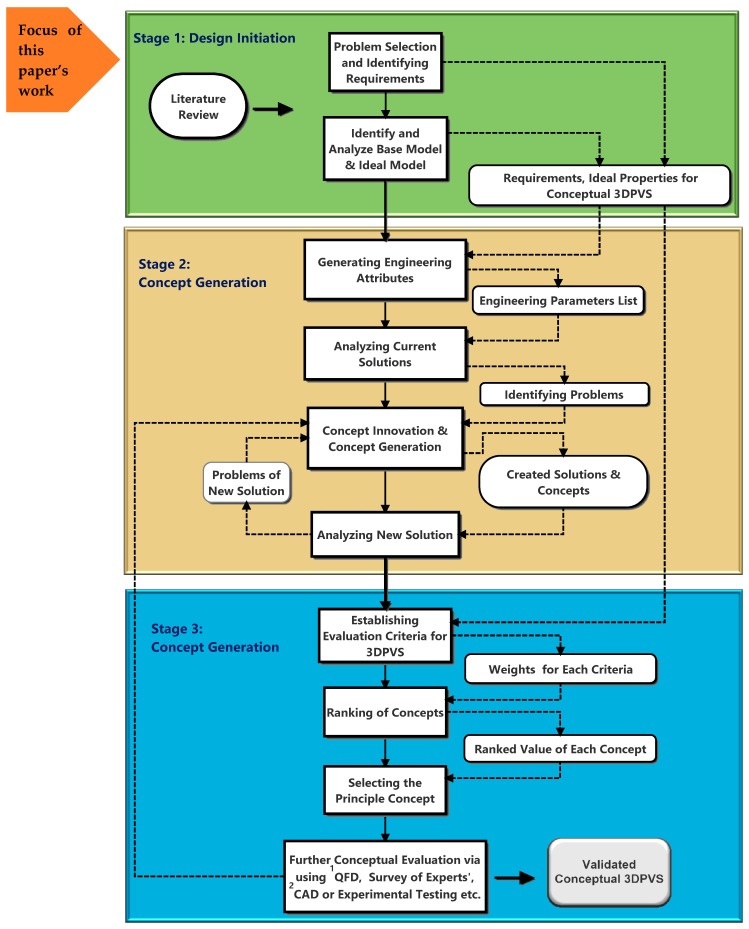
Illustration of the three-stage process for the development of the conceptual 3D printed vibratory scaffold (3DPVS). Note: ^1^ Quality Function Deployment, ^2^ Computer Aided Design.

Design initiation is chiefly aimed at information gathering, problem selecting, and requirement identifying. Since the acquired information can be used as the input for both the concept generation and evaluation, it is of significant value for any further product innovation. A slight mistake in this stage could trigger the domino effect in later design, which would make it hard to find appropriate inspiration to achieve innovation, and the design quality could spiral downward. Figure 1 shows the schematic of the three-stage process to develop the conceptual 3DPVS. For detailed explanation of each step, please refer to previous work [3]. In this paper, a systematic study on the first stage, namely the design initiation, will be the chief focus, which is expected to help thoroughly prepare the next two design stages in future.

### 1.4. Design Initiation as the Study Focus of this Paper’s Work

As introduced, design initiation is the preliminary task that designers need to focus on before participating in any further innovation processes. In terms of its scope, it includes two main aspects: firstly, a thorough literature review regarding the cell culture scaffold, 3DP, vibration, system evolutional laws, etc.; secondly, a good preparatory work gathering design requirements, analyzing the characterization of the 3DPVS, and generating a base model with which the design process starts, as well as indicating the ideal model of design, which is considered as the ultimate destination that the conceptual product should finally approximate. Previously to this paper’s work, the first task of design initiation has been conducted [1,3], and therefore, in this paper, we aim to focus on the remaining part, namely the second task of design initiation.

In this connection, four basic issues will thus be discussed in this paper’s context. Firstly, we will study several vital elements inside design initiation which will help prepare the general conceptual stage of the 3DPVS, after which the novel geometrical, mechanical, biological, and vibratory (GMBV) characterization of the 3DPVS will be analyzed, considering the GMBV as an unique design prospect of the 3DPVS compared with traditional scaffolds; thirdly, it comes to illustrating the base model of the 3DPVS and explaining the philosophy of the selecting process; finally, we will discuss the ideality of the scaffold, establish a possible ideal model of the 3DPVS, and briefly indicate the potential characteristics of such an ideal model.

## 2. Fundamental Aspects Initiating the Conceptual Design of the 3DPVS

As far as the fundamental understanding of the 3DPVS is concerned, four aspects will be chiefly focused upon in this section. First, we will talk about design initiation in terms of its problem selection and requirement analysis. Second, fundamental elements that constitute the 3DPVS as well as the direct or indirect relations between these elements will be studied. Then, why bone cells are preliminarily focused upon as currently proposed applications of the 3DPVS will be discussed, along with illustrating several most typical requirements of bone cell cultures. Fourth, the novel three-layer-based 3DPVS system will be discussed, and the corresponding demands during design for the three systems will be named as 3DPVS requirements, attributes, and components.

### 2.1. Problem Selection and Requirement Analysis

In a previous literature review [1], general information regarding research problems selected was mentioned after studying research limitation and gaps, with some possible future objectives put forward consequently. However, considering the very nature of the 3DPVS as the product to fulfil different kinds of requirements in external cell culture, how to select problems and determine ideal objectives could inevitably vary from one scenario to another; that is, it will be necessary to select different vibratory mechanisms and apply different 3DP tools to achieve functionality of a 3DPVS. Therefore, if those generic objectives generated in the literature review were the ‘trunk’ of a tree, then ‘branches’ would be 3DPVSs to be designed for different and specific scenarios. In this section, such knowledge of ‘branches’ will be studied, expanding the level of general problem selection and objectives of 3DPVSs into one which is detailed and ‘trunk into branch’.

Following this, the logical aspect is studying the fundamental purpose these ‘branches’ can and must fulfil. 3DPVSs, for instance, can be classified by whether they are designed for application of bone cells, other types of cells, or both. For 3DPVSs based on bone cells, they can be further divided into different products tailored for different requirements in bone cell culturing. This sequential categorizing possibly helps to understand the whole picture of the conceptual design process; that is, the previous literature review chiefly focuses on the first stage, conceptual design and generating solutions focus on the second, and utilizing the generic solutions of 3DPVSs in a specific case scenario of bone cells will be the focus of the third. 

On other hand, general limitations of previous 3D scaffolds and vibration mechanisms could be segmented into further detailed ones in order to fulfil the design requirements in different 3DPVS scenarios. In this connection, how to pass general limitations into specific ones when different case scenarios are concerned becomes an interesting issue. Consequently, this might bring about new research limitations or design problems which researchers did not anticipate earlier. Further, design objectives could vary from one case to another, according to what is uniquely required in the cell culture scaffold as well as the vibratory functionality. Similar to determining the newly selected problems from generic problems, the design objectives will be the result of two elements: the case requirements, involving selecting tailored cell culture scenarios, and the general 3DPVS objectives, which have been thoroughly studied via literature review. In brief, defining such requirements and objectives would be important tasks in design initiation for respectively conceptualizing base and ideal models.

### 2.2. Fundamental Relations of Elements that Constitute a Conceptual 3DPVS 

A justified conceptual 3DPVS is composed of several essential elements. The purpose of this section is to illustrate these elements, including geometric control, material composition, biological properties, and vibratory functionality, as well as the 3DP fabricability toward the future scaffold design. This could be achieved by penetrating deeper with the general knowledge obtained in the literature review [1] into detailed requirements gathered inside the later stage of design initiation. For the relations of these elements regarding 3DPVSs in different cell culture scenarios, possible pitfalls inevitably exist for designers. Understanding these relations would consequently accelerate the work in design initiation, as well as providing clarity toward establishing possible base and ideal models of conceptual 3DPVSs.

As discussed early, the conceptual objective of the 3DPVS is to evolutionarily transform a traditional passive 3D scaffold and externally attached ‘vibrators’ or ‘vibration mechanisms’ into a single organic unit. In design initiation, problems can be attributed to forms tuned with several basic elements which possess the most fundamental aspects constituting the conceptual 3DPVS. In this connection, solving possible problems resulting from undesired relations could be termed ‘internal’ problem-solving. Contrasting this is ‘external’ problem-solving. Issues such as undesirable cost of scaffold manufacturing or the low ability to conduct experiments on cell culture, among others, are of importance, but will not be the focus in the current design context, chiefly because ‘internal’ issues should be prioritized over the ‘external’ ones. In addition to this, another factor is about the current focus on conceptual design. Issues existing in future detailed design should not be prioritized by designers. As a result, the list of elements that designers need to concentrate on at the current stage is proposed as the five aspects presented in Table 1.

In brief, the basic relations of elements in design initiation, as discussed above, could be considered as the fundamental criteria that every possible 3DPVS needs to fulfil. Further betterment and detailed specification in addition to this could take place in future detailed design, which requires designers to address these relations in a more detailed, specific manner. Thus, checking whether the conceptual 3DPVS fulfills these elements in the first place is vital work for designers in the stage of design initiation, which gives insight to identifying the novel GMBV characterization of 3DPVS, as well as benefiting the future concept generation and innovation which follows design initiation.

### 2.3. Focusing on Bone Cell Culture and Summarizing its Typical Requirements

In order to understand the fundamental elements of a 3DPVS, this section selects bone cells as the preliminary application of a 3DPVS in this research context, as well as summarizing design requirements from general bone cell culture. This could help establish a 3DPVS base model and ideal model in design initiation, as well as providing a basis for utilizing 3DPVS design methodology in specific bone cell case scenarios. 

#### 2.3.1. Focusing on Bone Cells as the Preliminary Application of a 3DPVS at the Current Conceptual Stage

In this research concept, focus of application will be on bone cells most commonly cultured by traditional scaffolds, for example, osteoblast cells and (mesenchymal stem cells) MSCs. There are two reasons regarding this: Firstly, bone cells have been popular cell types, if not the most popular ones, selected for traditional scaffold engineering in cell culture. Much existing knowledge and experimental data therefore can be obtained to help designers explore novel 3DPVSs in an effective way. The second reason concerns the nature of the research context: it seems both practical and reasonable to focus on one definite group of cells when designing a new scaffold via a novel conceptual methodology. The success of this would easily lead to the design of 3DPVSs for less popular cell applications. This will keep the research objectives concentrated and the difficulty of design controlled at a relatively moderate level.

Since the application of 3DPVS is to be focused on bone cells, the basic knowledge of bone cells is useful to assist with the design process, as well as helping other designers who lack specific background in the bone-related biomedical area. In vivo, two types of cells, namely osteoblasts and osteoclasts, work actively in the bone generation process. Both cell types create new bone material at a slow rate. Osteoblasts are cells that build bones, and osteoclasts are cells that dissolve bones. Therefore, how to master the functionality of these two cells in vitro is believed as the foundation for research when it comes to controlling and regenerating bone cells in vivo [4,5,6]. In brief, for the design of 3DPVS, both in design initiation and the future potential case study, these two types of cells could possibly be the chief focus. 

When it comes to cell culturing environment, during design initiation, the 3DPVS is proposed to use similar cell culturing media to that applied by traditional bone cell culture scaffolds. Different cells would require different cell growth environments; the most suitable media for each cell culture usually is a challenging aspect and could be determined via experiments [7,8,9]. This could bring difficulty for designers who focus on scaffold innovation without a finalized scaffold at hand, since it is uncertain whether the previously applied culturing medium is adequate or not. In this connection, selecting a medium based on previous researchers’ experience is proposed as a solution. For the bone culturing environment, which is an important part of the base model, the following media could be selected, considering its high applicability in previous cell culture work and the bone-cell focus in this research context. 

A-modified Eagle’s medium (aMEM) (Bio-Whittaker, Belgium) containing 10–15% (*v*/*v*) fetal bovine serum (FBS, Gibco™, Thermo Fisher Scientific), 100–150 U/mL penicillin, and 0.1–0.2 mg/mL streptomycin (HyClone, UK), in a humidified atmosphere at 30–40 °C and 5–7% CO_2_ [4,10].

#### 2.3.2. Abstracting Typical Cell Culture Requirements for Bone Cells

After illustrating the elementary requirements for 3DPVS, requirements from general bone cell culture scenarios will be considered here, which would help accelerate the conceptual design process later, namely the concept generation and concept evaluation. In this connection, requirements in general bone cases for 3DPVS were abstracted from these studies [4,11,12,13]. Some of the most typical requirements of these can be summarized as follows:

- Focus on the cell culture aspects such as osteoblast differentiation and bone formation;
- Focus on the integrin- and BMP-mediated signaling pathways;
- Achieve definite activation of the angiogenesis of endothelial cells, which can help in vivo bone formation;
- Provide a tailored and specific biophysical microenvironment for bone cells;
- May use scaffolds as potential tools to regenerate damaged bone tissues;
- External stimulation such as selective heating could be required to generate definite stimuli on cells in some areas;
- Study spatial distribution in terms of ligands’ physiological aspects, including anisotropic vibration frequencies and isotropic vibration frequencies;
- Artificially created external bone cell culturing environments need to be flexible and dimensionally stable;
- Materials of a bone cell culture scaffold need to be biocompatible compounds;
- High-quality surface of culturing environment may be required, which leads to a better bone cell attachment;
- Bone cell culture scaffold demands an adequate pore size, which is necessary for bone cell migration, nutrient transport, and vascularization.

In addition to these requirements, other vital requirements for bone cell scaffold exist but will not be applicable in the context of 3DPVS design. Using one critical requirement of bone tissue scaffolds, for instance, which is regarding the identification and control of mechano-regulation on tissue differentiation by biophysical stimuli, it is important since mechanical integrity is maintained once the scaffold is implanted in vivo. The benefit of bone tissue scaffolds enabling in situ load-bearing is therefore valuable to the orthopedic field as current bone void fillers are non-load-bearing. However, for the external cell culture scaffold (ECCS) which is the focus of our research, this requirement is less essential because the ECCS, to which 3DPVS would belong, will initially be designed for cell cultivation without a further tissue-implantation process. This difference therefore could determine the different set of design attributes for different scaffold systems. 

### 2.4. Design Requirements, Attributes, and 3DPVS Subsystem Components

Viewed from the point of system evolution, every product can be defined as a system, which belongs to a wider product system called a supersystem and consists of component-based product systems termed subsystems [14]. In this connection, a 3DPVS system will contains a three-level system hierarchy, namely the 3DPVS supersystem, systems, and subsystems. The three types of required or necessary design elements constituting these three systems can be respectively termed as requirements, attributes, and components. These concepts will play a significant role inside the full design process and will be clarified in the following sections. 

#### 2.4.1. Categorization of 3DPVS Supersystem, Systems, and Subsystems 

To be specific, from the TRIZ (Theory of Inventive Problem Solving)-based systematic thinking, requirements during the entire process of design, surely including the design of a 3DPVS, can be divided into three layers. First is the term ‘requirements’: design requirements or requirements in design initiation that have been used in this research context will relate to the supersystem realm and indicate the very application of the 3DPVS, such as the requirements of cell culture, dynamic bone cell cultivation, and so forth. Second, following supersystem requirements come the requirements at the system level, namely the design requirements for the 3DPVS, including geometries, material composition, and biochemical properties, etc. To make the distinction clear and avoid ambiguity with the use of the term ‘requirements’ in the supersystem level, we will term this layer’s requirements as ‘attributes’ for 3DPVS design. After this, the third-level requirements will be concentrated on the components inside the 3DPVS, namely the subsystem requirements of the 3DPVS. Parameters such as the scaffold’s height, weight, vibratory frequency, and so forth could be the typical elements constituting this requirement list. In this connection, the requirements of this layer will be defined as ‘components’ or ‘subsystem components for 3DPVS’, to distinguish from the requirements of the higher two layers. 

#### 2.4.2. Structures of 3DPVS Requirements, Attributes, and Components 

**Figure 2:**
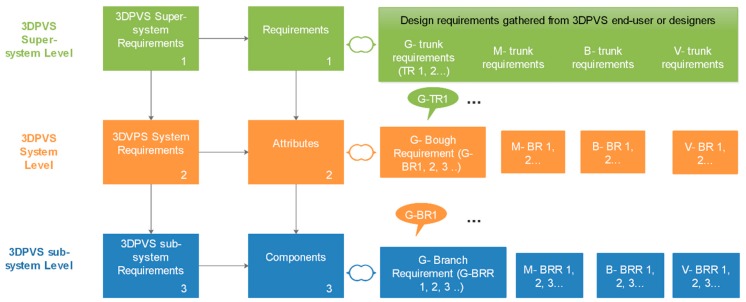
Illustration of the three-level requirements in 3DPVS design, namely ‘requirements’, ‘attributes’, and ‘components’. G: geometric, M: mechanical, B: biological, and V: vibratory.

A three-layer structure of demands of design for the 3DPVS system will be discussed in this part. The fundamental requirements (supersystem level) connect with the subordinate (system and subsystem) requirements in a certain definite way. Out of the subordinate requirements of the first order come the subordinate requirements of the second order, and so on. The construction of requirements, regarding the whole picture of general or specific requirements from the beginning of design toward its very end, can be compared with the construction of a tree. From the straight basic trunk, there come out boughs on all sides, which in turn divide and pass into branches becoming smaller and smaller, and finally are covered with leaves. The same process goes on in the construction of the leaves, in the formation of the veins, the serrations, and so on. Figure 2 shows the three-level requirements and their relationships regarding three vital elements in the design initiation phase, that is, requirements, attributes, and components. 

On other hand, the fundamental properties of 3DPVS consist of four aspects, namely the geometric, mechanical, biological, and vibratory (GMBV) characterization; in this connection, we could generate a structured list of input requirement, design attributes, and 3DPVS components, which will be analyzed using these four aspects in a similar way. This approach would help make the entire design process efficient and easily understandable. The establishment of innovative methodology of 3DPVS, which will be studied as a future task, could also benefit from such an approach, since the different levels of requirements mean more design details, which could increase the accuracy, especially in the stage of selecting innovation principles for conceptual generation.

As is often the case, requirements directly obtained from the end-users or initiating designers might be general, unstructured, and sometimes vague. The research objectives regarding technical aspects of the 3DPVS could be considered as technical requirements where general 3DPVS requirements at the system level need to be fulfilled. Attributes and components that respectively act as the role of ‘bough’ and ‘branch’, are of more significance when it comes to the conceptual stage of design, compared with the ‘trunk’, which is usually informed by general end-users. On other hand, further requirements in detailed design, namely ‘leaves’ requirements, can and should be focused upon after the successful finalization of the conceptual design. In brief, the work to structure requirements into the language that can be used by designers, for example, the language designers use when applying a methodology, as well as identifying the proper attributes and components inside the design process, is of significant value and will be illustrated in future design work. To conclude, the construction of 3DPVS requirement, attributes, and components has been focused upon in this section, following which we could introduce the GMBV characterization of conceptual 3DPVSs in a reasonable way.

## 3. Introducing the GMBV Characterization of Conceptual 3DPVSs

In the previous section, several elementary aspects regarding design initiation have been studied, and here, a detailed characterization of 3DPVSs, compared with traditional 3D passive or static scaffolds, will be analyzed, which is proposed to help understand the conceptual 3DPVSs with more clarity as well as benefit the process of establishing proper base and ideal models for 3DPVSs.

### 3.1. Basic Philosophy of GMBV Characterization

As discussed earlier, in traditional static or passive scaffold engineering, three characterizations are usually considered when studying or designing properties of a scaffold. They respectively indicate the scaffold’s geometric controls, mechanical properties, and biological functions [15]. These characterizations could be utilized for studying and designing future scaffolds, such as 3DPVSs, as well, chiefly because the essence of the scaffold, being static or vibratory, basically remains the same in these three aspects when it comes to culturing cells via a scaffold. The only difference for 3DPVS is that vibratory functionality, or vibratory dynamicity, would be a prioritized design aspect in the conceptual stage of 3DPVS, since this is the very innovative point initiating the design. Therefore, it is worthwhile to introduce a new direction for the systematic design of 3DPVSs. In this connection, the characteristics, namely the elements and parameters, which both generic or specific 3DPVSs might need to possess during various cell culture scenarios, would be made into a new category. This category can be termed as ‘GMBV characterization’, consisting of four properties of novel scaffold design, that is, the geometric properties, mechanical properties, biological properties, and vibratory properties. 

On other hand, for both base models and ideal models of 3DPVS, designers need to consider these four characteristics. The two sets of models respectively represent the base point of the scaffold with its related environmental issues to initiate the design and the ideal end point where the 3DPVS is considered as highly optimal. In this connection, the design philosophy from base models into desired objectives in terms of these characteristics tends to be consistent. Parameters and specifications of base models, design objectives, and ideal 3DPVS will be studied and evaluated separately inside the newly put-forward GMBV characterization. This could make the design process more structured, clear, and specific when designers analyze issues in the conceptual stage of 3DPVS.

Following the GMBV analysis, new understanding regarding base and ideal models occurs. In terms of base modeling, it can be established from analyzing the GMBV characterization. For instance, designers will choose proper 3D static or passive scaffolds with specific G, M, and B properties, as well as selecting possible vibration mechanisms as the V properties to start the design. Inside each of the G, M, B, and V characterizations, a relatively large group of parameters or properties will be related, and many of those should and can be the design focus in future stages of detailed design. Considering that the priority of this current research lies in the conceptual development of 3DPVS, the parameters which are specifically tailored for conceptual stage will be studied and utilized. Key information regarding this will be discussed afterwards.

### 3.2. Geometric Properties (G-Characterization)

Geometric control could be considered as vital in both traditional 3D static or passive scaffold and potential 3DPVSs. CAD designs of scaffold chiefly deal with the geometric aspects.

#### 3.2.1. Geometrics selected for the Base Model of Conceptual 3DPVSs 

**Figure 3:**
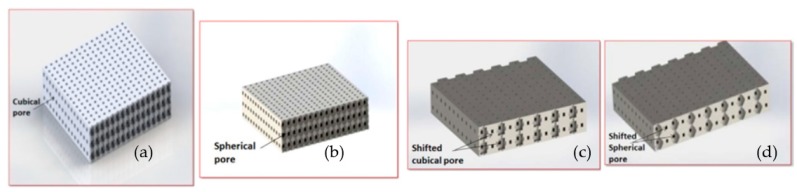
Four geometrics of porous scaffold that could be applied as base models. (**a**) Cubical pore model; (**b**) spherical pore model; (**c**) shifted cubical pore model; (**d**) shifted spherical pore model.

As studied in the literature review [1], there have been a wide range of geometrics applied in traditional scaffolds and to tailor geometrical control with current research aims; the four basic geometrics which are most commonly used for bone scaffolds, namely the cubical, spherical, shifted cubical, and shifted spherical porous geometrics, could be used for 3DPVS conceptual design. These four typical geometrics will be considered as the starting point for the geometrics of the base model, due to their ease of design allowing new features to be easily added. In terms of the porosity range, it generally varies between 30% to 80% for culturing bone cells [16,17]. Figure 3 illustrates the four geometrics which would possibly be used as the base geometrics in the conceptual design of 3DPVSs.

#### 3.2.2. Basic G-Characteristics Selected for 3DPVS Design

Following the discussion of the four geometrics, G-characteristics will be defined through parameters or properties related with geometrics. To be specific, following aspects, each containing a pair of opposite properties, will possibly be the most vital factors during the geometric design of 3DPVSs. These paired sets could be used by TRIZ in future conceptual design. 

Volume (high–low), geometric complexity (high–low), pore size (big–small), pore randomness (high–low), surface roughness (high–low), geometrical stimulation (high–low), geometric precision (high–low), wall thickness/beam diameters (high–low)

Besides establishing basic geometrical perspectives, each of these parameters or aspects could be used to judge the ideal level of the 3DPVS. Taking the surface roughness, for instance, if the desired value of the roughness property needs to be high in order to be ideal based on some design criterion established, its low values will be considered as nonideal, and vice versa. In this connection, for studying ideal models, the G-based ideality will be calculated by the proposed properties, or part of them, as listed above. 

In terms of the geometric characteristics of a scaffold related with 3DP properties, studied from [18,19,20], several aspects might be important: flexibility of structural design (high–low), fabricability for free-shaped structures (high-low), minimum feature size (large–small), structure minimal precision (high–low), fabrication time (long–short), width of printed line (high–low), and CAD compatibility (high–low).

### 3.3. Mechanical Properties (M-Characterization)

Mechanical control of 3DPVS is proposed to be similar with that of traditional 3D static or passive scaffold, and material composition is the essential factor affecting different means of mechanical control. 

#### 3.3.1. Materials Selected for the Base Model of Conceptual 3DPVSs

To fabricate scaffolds for bone tissue engineering, polymers, ceramics, and their composites have been widely used [19]. Although not every material selected for the base model here will be used in future 3DPVS design, it is useful as a starting point at design initiation. 

Several more popularly used polymers could be used as materials for the base model. These materials provide proper mechanical properties for the scaffold as well as being fabricable by different 3DP systems. These materials are naturally static and passive. Comparing these materials with the novel materials tailored for vibratory functions of 3DPVSs could be important during the design process. In brief, the following materials used for traditional 3D scaffolds will be used as the starting point base-model materials toward the ideal-model materials of 3DPVSs.

- Polyglycolic acid (PGA)
- Polylactic acid (PLA)
- Polyethylene glycol (PEG)
- Polyamide (PA)
- Polycaprolactone (PCL)
- Polyurethane (PU)
- Hydroxylapatite (HA)

#### 3.3.2. Basic M-Characteristics Selected for 3DPVS Design

Basic characteristics regarding mechanical properties selected for the base model of 3DPVSs include, but are not limited to, the following:

Systematic controllability of properties, SCP (high–low); anisotropic level (high–low); scaffold syntheticity (high–low); material availability (high–low); mechanical stability of structures, MSS (high–low); chemical stimuli (proper–improper); applicability to cell study (high–low); surface biochemical functionality (high–low).

For evaluating the level of ideality regarding mechanical control of 3DPVSs, the philosophy is the same as what has been discussed in the G-characteristics section. In brief, much of the contents would only be used in future detailed design after the conceptual development stage. 

### 3.4. Biological Properties (B-Characterization)

When it comes to biological properties of 3DPVSs, there is a chief difference compared with the G and M factors as discussed previously. For biological properties, they are usually approached indirectly by designers; that is, biological properties tend to be the resultant byproduct of many other factors of the scaffold on cultured cells. Both the characteristic variation of G and M factors could result in different properties of B-characterization. On other hand, the quality of biological properties also can be used as criteria through evaluating the idealism or ideal functionality of the scaffold. 

#### 3.4.1. Biological Properties Selected for Base Models

In terms of the basic B properties of proposed 3DPVSs, they are currently considered as predominantly the same compared with the biological properties of traditional 3D passive or static scaffolds. Several most vital aspects can be summarized as follows:

- Can help cell proliferation;
- Can help cell differentiation;
- Can help cells remain 3D-shaped;
- Can help cells remain in positive contact with other cells;
- Can help achieve some required cell functions that differ from one case scenario to another.

#### 3.4.2. Basic B-Characteristics Selected for 3DPVS Design

Cell culture characterization regarding biological properties has been a sophisticated issue. From literature study [21,22,23], possible characteristics, with opposing values for each property, could include, but are not limited to, the following:

Cell survival rate (high–low), cell proliferation (high–low), cell viability (high–low), stiffness of the environment (high–low), stem cell differentiation (high–low), cell morphology quality, CMQ (high–low), topography of cellular environment, TCE (high–low), spatial patterning of ligands, SPL (high–low), length of ligand (high–low), SPL cell morphology (high–low), efficiency of generating extracellular matrix (ECM) (high–low), presence of biomechanical cues (high–low), experimental model maturity, EMM (high–low), integration of different stimuli—physical, biochemical, etc. (high–low), growth factors (low–high), signaling and adhesion molecules, SAM (high–low), cell behavior (high–low), stiffness of the environment (high–low), dynamicity of the environment (high–low), cell–cell interaction, CCI (high–low), cell–matrix interaction, CMI (high–low), cellular force generation (high–low), intracellular structure quality (high–low), cytoskeleton (high–low), cell/matrix adhesion (high–low), natural 3D recapitulation (high–low), biomolecule immobilization (high–low).

For conceptual design of 3DPVSs, it is not necessary to focus on every property listed. That is, the most basic properties would be selected for the conceptual stage and other properties would be most likely focused upon in the later detailed design stage. How to prioritize different basic properties of a scaffold is therefore important for designers. 

### 3.5. Vibratory Properties (V-Characterization)

For the base model, vibration or vibratory dynamicity mechanisms, which was studied in literature review, will be the start point for conceptual design. At the design initiation stage, the traditional 3DP scaffolds placed inside the dynamic bioreactor, as well as the 3D scaffold attached to external vibrator shakers, could be selected as the base vibration model. 

#### 3.5.1. Vibratory Dynamicity Selected for Base Models

Based on the study, three vibration modes can be considered as the starting point for conceptual design of 3DVPSs.

- Mechanical vibration through external vibrators
- Bioreactor-based vibration
- Subtle vibrations, including ultrasonic vibration, vibration using piezoelectric devices, etc.

#### 3.5.2. Basic V-Characteristics Selected for 3DPVS Design

There are several criteria establishing 3DPVS vibratory dynamicity. For the 3DPVS objectives of this research, they have been discussed previously, and integrated self-vibratory functionality of the scaffold—or the capability of the scaffold to actively generate vibrations—would be considered as one of the most vital elements for 3DPVSs. In this connection, several important characteristics need to be summarized, with some characteristics to be further explained for design initiation.

Vibration mechanism applicability (low–high)^1^; vibration frequency range (low–high)^2^; frequency accuracy (low–high); vibration localization level (low–high); vibration feasibility for required cell culture (low–high); vibration stimulation intensity (high–low)^3^, vibration three–dimensionality (high–low) ^4^. ^1^ Whether it can be applied on the base model of a 3D static scaffold; ^2^ Range of the affordable frequencies between minimum to maximum values; ^3^ Different dynamic cell cultures require different stimulation intensity; ^4^ 3D vibration generates at three directions namely x-, y- and z- axis. 

In previous sections, we have illustrated the GMBV characterization for 3DPVSs as well as some issues related with establishing base models. Inside each of the four sets, a list of most frequently used parameters and attributes by designers have been illustrated, which could be used as criteria to judge the ideality level of a 3DPVS. For each parameter, a pair of contradictory definitions or values has been identified, with one indicating the parameter as ideal and the opposite other as undesirable. That is, using porous size, for instance, it is defined as ‘small–big’, and for a definite design case where the pore size of the scaffold needs to be larger, values of ‘big’ will be considered as ideal and values of ‘small’ correspondingly as undesirable. Following this philosophy, the level of ideality can in turn be judged by evaluating whether the real value of the attribute stands closer to the ideal or undesirable extreme inside this ‘contradictory set’. Proper ideality level and ideal model of the 3DPVS will possibly be established. In addition, such a ‘contradictory set’-based approach would tailor the TRIZ-oriented design methodology, which will help achieve the concept generation after the design initiation phase. This makes the design process effective and reasonable. 

To conclude, GMDV characterization has been introduced in this section, and it aims to provide designers with a systematic strategy in analyzing, focusing, and categorizing different design aspects of 3DPVSs, which usually have been considered as complicated for traditional scaffold designers. Scaffold innovation in this connection would follow in a relatively natural way. 

## 4. Establishing the Potential Scaffold Base Model for Conceptual Design

In the previous sections, several vital issues in design initiation have been studied with bone cells as the focused application for the conceptual 3DPVS. GMBV characterization, which copes with the design issues regarding geometric control, mechanical control, biological properties, and vibratory dynamicity, has been put forward. In this section, we will establish potential base and ideal models for conceptual 3DPVSs, and this is a vital task in the design initiation stage.

### 4.1. Significance of Identifying the Base Models

In terms of the significance of base model, it is the starting point of where the concept generation comes. In this study, the practical use of the base model is to anchor the design into a rather practical realm, which briefly includes two aspects. First, what will have been generated as innovation concepts or conceptual solutions in the concept generation stage need to be applicable, and at least reasonable, to the base model analysis, otherwise the novel concepts would simply be theoretical and inappropriate for practical use. Second, base scaffold models that have been applied, studied, or investigated by previous research at a relatively mature level are preferable; that is, the selected initiating scaffolds should not be something new, which will ensure that the majority of their characteristics and effects on cell culture are already certain, known, and accessible. 

In this connection, one key job of using base models inside the conceptual design is to extract a set of characteristics, or parameters, from these selected scaffold models where the conceptual innovation can start. Base scaffold model, therefore, can be logically defined as a basic version of a 3D static or passive scaffold product, on top of which alterations, additions, or enhancements can be designed; it could specifically refer to typical or easily accessible 3D cell culture scaffold models experimented upon or utilized under traditional vibrations, namely mechanical vibrations, external vibrators, and so on. In the next section, knowledge regarding base models for 3DPVS conceptual design will be discussed, and three base models corresponding with three 3DP systems can be established. After this, several aspects regarding GMBV characterization, which could play the bridging role between base and ideal models, will be studied. 

### 4.2. Selecting the Trinity of Base Scaffold Models through 3DP Categorization

In design initiation, base scaffold models are needed. Quality of base models selected and the philosophy behind selecting them is of significance for the whole conceptual design that can follow reasonably afterwards. One important question logically appears: what is the basic philosophy to select the base model(s)?

As studied, 3DP plays a core role for novel 3DPVSs, because 3DP is the proposed bridging technology between traditional and novel bone cell culture scaffolds, as the proposed 3DPVS will be fabricable by 3DP methods. In this connection, the base model can be logically classified into three categories corresponding to the three classes which match up with the three-group categorization of 3DP tools. In other words, base scaffold models for the 3D static or passive scaffolds which have been fabricated or approached through laser-based 3DP, nozzle-based 3DP, and droplet-based 3DP technologies could be selected. To make the design efficient, in each 3DP category, a relatively popular, easily applicable 3D scaffold will be selected as the starting point toward developing it into a possible ideal scaffold model. 

Considering 3DP systems and other aspects for conceptual design, base model(s) would be further selected and determined through the following criteria. Firstly, the base model needs to be 3DP-fabricable. Scaffolds fabricated by conventional tools tend to be inappropriate. Secondly, the base model needs to have been previously applied in cell culture, especially in bone cell applications, since the application focus of 3DPVSs at the current stage is focusing on bone cells. Novel scaffolds not utilized for external bone cell cultivation will not be selected. Thirdly, base models need to be potentially upgradable and modifiable; that is, their characteristics regarding geometric, mechanical, biological, or dynamic properties should have flexibility to change according to different design requirements. In brief, characterization of the base model scaffold ought to be simply and flexible. In the following sections, three sets of base models corresponding to the trinity of 3DP systems will be illustrated. These models will be used as the starting point toward achieving the ideal modes of 3DPVSs. 

It is also noteworthy that although base models will be categorized into three classes in accordance with the trinity of 3DP methods, the ideal scaffold model could be beyond this classification, because that ideal model of a 3DPVS could either be fabricated by the 3DP which initiated the base model inside the same category, or could be achieved by another 3DP method, or even by a hybrid 3DP system which potentially integrates different 3DP fabrication methods. After generating basic solutions by TRIZ, each solution could be ranked based on the feasibility of 3DP methods; that is, the more feasibly and practically the solution can be achieved by definite or multiple 3DPs, the better its ideality would be. 

#### 4.2.1. Laser-Based 3DP Base Model of Scaffold 

**Figure 4:**
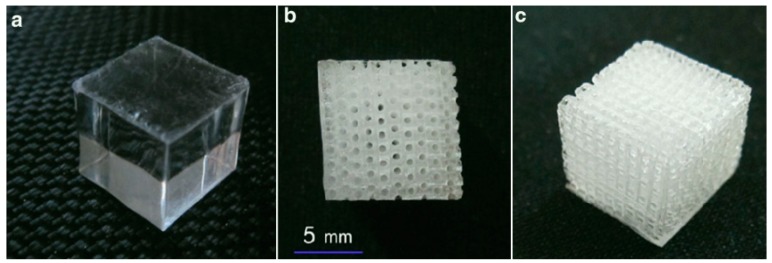
Bulk cubic specimen before 3D printing (3DP) laser processing (**a**). A front view of the fabricated PMMA scaffold showing regular pattern of holes (**b**). An isometric view of the scaffold showing inward 3D network (**c**).

Laser-based scaffold will be used as the base model. One promising laser-based 3DP for this model can be for example ProJet 1200, which is a recent stereolithography (SLA) laser-based 3D printer. Figure 4 below shows a typical 3D laser drilling Poly (methyl methacrylate) (PMMA) scaffold used for bone regeneration [24]. It generally has foaming holes with diameter range around 500 μm and depth about 10-15 mm, center distance about 0.5–1.5 mm, achievable porosity at 40–75%, and compressive strength at about 20–60 MPa [12,24,25].

The reason to select this scaffold sample as the base model is that different scaffolds with increasing levels of porosity can be fabricated using same method, and different laser-based techniques easily produce similar products. In this connection, there is no need to make fixed parameters regarding the scaffold’s overall size, pore size, and inner mechanical strength regarding the base scaffold model in this category.

#### 4.2.2. Nozzle-Based 3DP Base Model of Scaffold 

**Figure 5:**
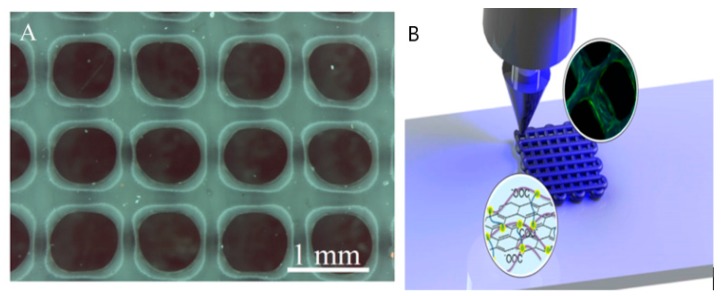
PTMC scaffold created by a 3DP nozzle system (**A**) and illustration of printer nozzle during fabrication (**B**).

Nozzle-based 3DP system, as discussed, plays a significant role for the general scaffold and will be so for the novel design of the 3DPVS. A typical scaffold model fabricable via nozzle-based 3DP will be selected as the base model of the 3DPVS. To be specific, Figure 5 shows the 3D-printed poly (trimethylene carbonate) (PTMC) scaffold, made of UV cross-linkable graphene/poly (trimethylene carbonate), with 1 × 1 cm dimensions and a pore size of 500−1000 μm, printed using a KIIM Bioplotter. Single- to multilayered (up to 7 layers) PTMC scaffolds can be effectively fabricated by this 3DP method. Accessible mechanical strength of this scaffold model is usually between 3–8 MPa [18,19,26]. The reason for selecting this scaffold as base scaffold model is that the compatibility of such a composite scaffold and its equivalent 2D films have been thoroughly tested with human MSCs, and the evaluation of the effect of such a scaffold on MSCs under osteogenic differentiation conditions has been considered as mature. In this connection, it might be relatively easy and convenient to develop such a base scaffold into the required bone cell culture 3DPVS. 

#### 4.2.3. Droplet-Based 3DP Base Model of Scaffold

**Figure 6:**
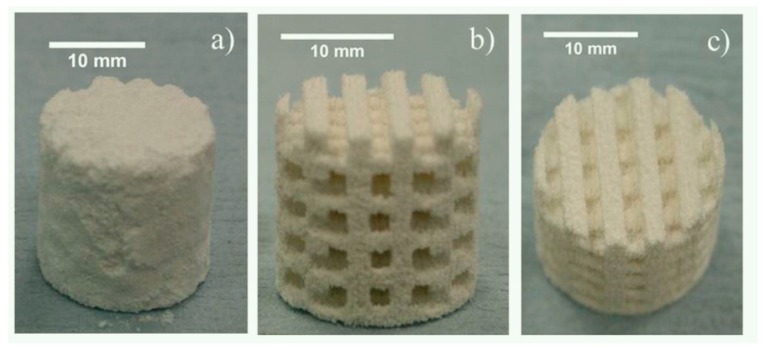
(**a**) 3D Inkjet-printed scaffold as removed from the build bed; (**b**,**c**) depowdered scaffold.

In the base scaffold model of this 3DP categorization, one typical 3DP droplet-based HA scaffold will be selected. This scaffold product typically has matrix structures with an interconnected system of pores ranging in size from 0.5 to 500 mm, with HA content (wt. %) related with Poly(vinyl alcohol) (PVOH) precursor powders between 50–60%, pore channel diameter between 1400 and 1800 μm, and micropore size between 10 and 60 μm, with numerous topographical irregularities implying a high degree of roughness. Porosity of this scaffold implies an average of 50–60% volume utilization, and the average compressive strength stands at 0.3–0.8 MPa, which is relatively lower compared with the value for scaffolds made by laser and nozzle 3DP systems [6,27,28]. In addition, bioresorbable polymeric scaffolds with specified architectonics for tissue engineering can be easily achievable from this base model. Typical materials used are solutions of polylactoglycolide in tetraglycol with their subsequent solidification in aqueous medium [6,27]. The chief reason of selecting this droplet-based scaffold as the base model is due to its relatively larger size compared with scaffolds in laser or nozzle categories. This means that the 3DPVS developed from this base model can fulfill definite requirements of cell cultures when the cultivation of or experimentation upon a large group of cells is necessary. An illustration of the scaffold as well as its construction in different stages of the 3DP process is shown in Figure 6.

## 5. Discussing the Ideality of the Scaffold and Establishing Ideal 3DPVS Models

The ideal destination of the 3DPVS, fulfilling its role in the scaffold’s evolutionary ladder, is considered to be the ideal model. Ideal model is also an organizational improvement model that serves as a roadmap for initiating, planning, and implementing improvement actions over the 3DPVS design process. For different stages of 3DPVS development, namely the design initiation, conceptual design, and detailed design, ideals or ultimately desirable goals would inevitably be different. That is, the ideal model for the 3DPVS to achieve at the conceptual design stage would be different, distinctly or slightly, compared with the ideal model to arrive at in the detailed design stage. Two sets of ideal models therefore exist. In this context, the ideal scaffold models talked about here would refer to the final ideal 3DPVS product after the detailed design. For the research objectives discussed in the literature review, they have referred to objectives that will be approached during the conceptual design. In brief, a gap will inevitably exist between the conceptual 3DPVS as research objectives at the conceptual level and the ideal 3DPVS as a final product in reality.

### 5.1. The Levels of Ideality Regarding GMBV Characterization 

For general ideality issues in an engineering system, laws of system evolution (LSE), especially the ‘law of increasing degree of ideality’, could help provide a clear vision. Elements of time, cost, space, and manufacturing all could contribute to the ideality levels of designs and products to some extent. In this connection, ideality of the scaffold will be based on this knowledge. Following this, we would speak about the ideality aspects specified with the 3DPVS. 

GMBV characterization, which was put forward previously to understand the basic properties of the 3DPVS, can be used to understand the ideality of the 3DPVS and the related ideal model(s). That is, designers can establish four levels of ideality corresponding to each of the four properties inside GMBV characterization. This will be the fundamental point where ideality can be basically judged. Besides this, another proposal could be that the interrelationship of each pair of different properties would contribute to the ideality level as well. In this connection, constructing the six pairs of relations, namely the G–M, M–B, B–V, G–V, M–V, and G–B relations, could bring the ideality evaluation into a more sophisticated level. Furthermore, putting three relations together, i.e., G–M–B, G–M–V, G–B–V, and M–B–V, it will give four sets of trinity relations, which could in turn determine some aspects of the ideality level of the 3DPVS. In this connection, a total amount of fourteen ideality relations could exist if designers would like to evaluate the ideality level of the 3DPVS in this philosophy. The ideal models of the 3DPVS could therefore be defined as the models that fully satisfy the ideal requirements or specifications inside each of these related aspects. In other words, the ideal model of the 3DPVS would necessarily require each of the G-, M-, B-, and V-characterizations to be ideal, as well as ensure the that different possible relations derived from GMBV characterization are ideal. In reality, this is inevitably unachievable, but it could be considered to the maximum by designers, and this also represents the very process of becoming ideal from nonideal. 

Detailed work of establishing and evaluating the ideality levels could only be thoroughly discussed in future stages of concept generation and evaluation, as well as the stage of detailed design. Gaining a full picture of conceptual specifications and the specifications in detailed design will therefore be of high necessity. In future conceptual design work, the relative importance of the G-, M-, B-, and V- characterizations and the combined properties, as well as their resulting contradiction level, would be judged and calculated. Experts’ opinions on this, due to the cross-domain nature of 3DPVS design, can be considered as useful. 

In the design initiation phase, we aim to give a brief proposal regarding the ideality evaluating mechanism. The ideality level for the 3DPVS system is to be determined in reverse ratio to the level of contradiction or conflict, which inevitably exists in each of these relations, as discussed above. Knowledge regarding contradictions will be studied in the later section on TRIZ-based methodology. Basic philosophy could be that the lower the level of contradiction or conflict inside the 3DPVS system, namely the supersystem-, system-, and subsystem-level GMBV properties and potential relations among the properties, the higher the level of ideality that can be expected. Calculating the sum of ideality value for each separate aspect would give a result of the overall ideality of the 3DPVS as an integrated product. 

**Figure 7:**
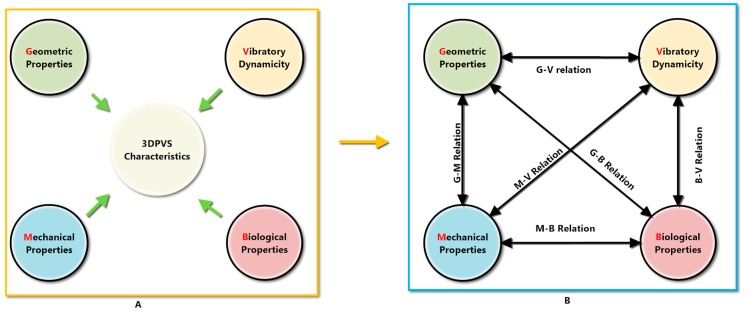
Four characteristics in 3DPVS design (**A**), and pairs of relationships regarding 3DPVS ideality modeling (**B**).

Following concept generation, designers in concept evaluation will create criteria, directly or indirectly calculate the contradiction or conflict level, and analyze the adequacy of generated solutions or concepts in a definite case scenario. To establish ideal models of a 3DPVS, the GMBV characterization will chiefly be analyzed, ensuring that the ideal scaffold model is neither too complicated that designers could find it difficult to set objectives, nor too simplified such that following the conceptual stage, the scaffold could lose its innovative value. Figure 7 below shows the relations inside GMBV characterization which could be used as one approach for both setting and judging the ideality level of a 3DPVS. 

### 5.2. Steps That Help Identify Objectives and Ideal Models

Several questions can help identify objectives as well as establish the ideal model of the design. These can be considered as decision support, which help designers analyze the aims to achieve and the possible hindering forces. With a hindrance detected, an ideal condition can usually be determined to establish ideal model. This strategy could help define both the objectives in conceptual design, as well as the objectives of 3DPVS as the ultimate ideal model. A seven-step approach could be summarized, with an example inside 3DPVS design used for better illustration.

- First, designers need to identify the ultimate destination of the design; using one chief goal of the 3DPVS as an example: to enable the scaffold to generate required vibrations which could help in specific bone cell cultures.
- Second, identify the ideal solution. The identification could be that the scaffold becomes self-vibratory under definite stimuli or means. Evolution in this context means a mature change, not the modification of external traits of the traditional scaffold.
- Third, identify the obstructions hindering us to attain an ideal solution; for example, the required stimuli or means that remain yet unknown to designers. Designers thus lack the very inspiration where the innovation could start with, let alone mastering the further elements in design process.
- Fourth, identify the elements that constitute the obstructions. In this connection, one chief cause could be related to material composition, since the materials applied have only been used for static cell culture conditions.
- Fifth, identify the ways of decreasing or eliminating the effects from the obstructions, directly or indirectly. That is, one possible direction is to change the material composition of the scaffold from using traditional static materials into smart materials, which could possibly be dynamic under definite conditions.
- Sixth, identify the resources can be helpful regarding this change. Resources chiefly refer to technical aspects such as the available science in biological, biochemical, and material realms. Expertise of technicians and a sufficient amount of knowledge is always necessary for a successful innovative design.
- Seventh, check whether some existing tools or methods in other areas could be useful in support. This might include design methodology and its related tools. Resources of technology alone are usually not enough, though they may cover the scope where the design focuses. To help designers achieve the focus, proper methodology is therefore considered as important and indispensable.

Following this strategic thinking, objectives in step two were determined as the summarized ideal model for conceptual design, where it focused on limitations and defects as obstructions, as discussed in step three above. In this connection, ideal models of the 3DPVS, after future detailed design, can be the ultimate extension of the ideal model settled at the conceptual design stage. Understanding this, as well as combining the knowledge of GMBV in terms of the multi-relation ideality, we could reasonably establish the ideal model for the 3DPVS product. 

### 5.3. Ideal Model of the 3DPVS and Discussing the Ideality of the Design Process

The ideal model of the 3DPVS means the ideal present-to-future 3D vibratory scaffold model. The ideal 3DPVS model describes the scaffold product as being fully self-vibratory under definite vibration mechanisms and able to generate the required vibration output, such as definite frequencies, by its whole structure or parts thereof. The ideal model can be fully fabricable by one or several of the existing 3DP systems. In addition to this, the ideal model should be constructed by materials fully fulfilling the mechanical as well as the vibratory or dynamic properties expected for a 3D scaffold in different cell culturing cases. In this way, scaffold constructs would be enabled with exact mechanical function as desired, and cells cultured inside could receive exact vibrations from the scaffold with the full accuracy required. The geometrics can be flexibly large or small to suit different cell culture environments, and therefore, the cell application range should be much larger than the current focus on bone cells. 

As far as this ideal model is concerned, there is little possibility to fully achieve it in either the present or future. However, this description gives a partial indication of the ideal future 3DPVS in terms of its direction of development. For a practical ideal solution or ideal model in design, it has value as a reference for comparing as well as understanding where the current design status stands. In brief, the ideal model of 3DPVS could only be infinitely approached during the design process while it is yet unachievable. 

#### 5.3.1. Technical Characteristics of the 3DPVS Ideal Model

In this section, the ideal model of the 3DPVS in terms of its technical characterization at the current conceptual development stage will be discussed. The core idea revolves around the GMBV characterization at the conceptual level, as well as the basic cell culture requirements in the bone scenario. As said earlier, for the required conceptual 3DPVS in definite cases, its ideality level could be basically estimated through analyzing the contradictory values of each pair of parameters inside the G-, M-, B-, and V-characterization. For example, ideality in geometric properties will inevitably include the ideal pore size, which would manifest itself at the conceptual level. If pore size is required to be relatively small, then ‘small’ would be considered as ideal and ‘large’ would be nonideal. Then, to what extent it is ideal or nonideal will be judged specifically for the given case scenario. A scoring method could be necessary when doing this. 

On other hand, the ideal model of the 3DPVS in detailed design tends to have more specific, definite, and accurate value regarding each parameter inside the GMBV characterization. In other words, the detailed specification of each parameter during detailed design could put forward the ideal 3DPVS model with each of the selected parameters being ideal and definite. For example, pore size for a 3DPVS could be set as an precise figure in the micrometer range. This figure could be approximated by designers in detailed design, while it is impossible to make the value of the real product absolutely the same as that of the ideal model. That is, the ideal model of the 3DPVS at its completion would indicate that the parameters or properties regarding all the elements identified by designers will be ideal and flawless. 

At design initiation, only a general introduction of ideality can be summarized. The ideal model of the conceptual 3DPVS is proposed to contain, but is not limited to, eight aspects. It is worthwhile to note that the following description of the ideal model will only be concept-based, without the detailed specifications, such as the exact pore size the scaffold needs to have, the exact vibratory frequency the scaffold needs to generate, and so on. The information of this could only be accessible in the stage of detailed design, or in the situation when the definite case scenario is accurately provided by the end-user. In brief, the description of the properties endowed by the ideal model of the conceptual 3DPVS is as follows:

- Ideal model should be applicable to different cells that can be cultured by traditional scaffold engineering systems and provide more beneficial effects over the traditional scaffold system;
- Ideal geometrics should refer to ideal interconnected porous networks or structures that allow for tailored cell growth and movement of nutrients and metabolic waste, etc., and newly designed geometrics should be in tune with the vibratory functions newly applied by the 3DPVS;
- Ideal model scaffold’s fabrication materials should be completely nontoxic, biocompatible, and potentially dynamic, as well as having highly appropriate effects regarding mechanical and biological control in different cell culture case scenarios;
- If the ideal model is designed as biodegradable, it would refer to a definite and accurate controllable level of degradation so that the scaffold can be replaced by bone cell mineralization in vivo. However, this ideal property is not necessarily required when the scaffold is merely used for external cell culture or cell study purposes;
- Ideal biological control can fully encourage the required cell attachment, proliferation, and other biological processes;
- Ideal model of the 3DPVS should have evolved completely from the traditional static or passive scaffold into the novel vibratory scaffold. It can be used as an ideal 3D static scaffold as well when the vibratory function is switched off; that is, the ideal 3DPVS model should fully replace the ideal model of the traditional 3D scaffold;
- Ideal model requires its dynamicity to be fully capable of generating tailored vibrations; for instance, vibrations in different areas of the scaffold localized to cells cultured inside, and the range of accuracy for the vibrations, especially the vibrating frequency, should possibly reach microscopic levels (from nanometer to centimeter);
- Ideal model requires the designed 3DPVS to be fully fabricable by one or more existing 3DP systems, endowed with all its properties and characterizations from the computer-aided design process.
- Ideal 3DPVS model has strict requirements on the relations between elements of GMBV characterization; that is, zero or minimum contradiction should occur between each pair, and the element in each pair functions in an ideal and perfect way without hindering the other.

Regarding several most vital target values of properties, several indications should be summarized before conceptual design, which might include: open porosity (50–90%); pore body diameter (dozens to hundreds of μm level); pore throat size (usually below 100 μm); surface roughness (dozens to hundreds of nm level); osteoblast proliferation rate: 15–25 h (doubling time); mass transfer of nutrients (cellular/substrate parameters), etc.; compressive strength (0.1 to 100 MPa level); stiffness (Young’s modulus) (2–10 MPa); vibration (from micro to macro levels), etc. However, these figures would be an indication instead of definite answers as to what they should be for future 3DPVSs. 

In this section, the idea model of the 3DPVS has been described, with its definition being the future scaffold concept/product fulfilling the entire criteria mentioned above. On other hand, if the final goal for scientists would be toward achieving the ideal model of the 3DPVS, then one condition that must be fulfilled is that the very design process for achieving the 3DPVS might also need to be ideal. That is, if a design process contains defects or problems, the designed product could potentially never be ideal. In other words, with an inadequate design methodology or roadmap, the final product must have limitations compared to the ideal goal. Therefore, knowledge regarding the ideality of the design process, such as methodology or design tools that could be potentially used for 3DPVSs, can be of value, though it is sometimes even harder for designers to invent a better-quality process than inventing a novel product.

#### 5.3.2. Ideal Design Process That Helps to Achieve the Ideal Scaffold Model

After illustrating the ideal model with its possible quality, characterization, and conditioning that the ideal 3DPVS might need to fulfil, in this section, we will talk about several key aspects regarding the ideality of the design process which will help designers achieve the ideal model from the base model. 

Traditional methodology for designing a 3D scaffold chiefly includes the CAD design. The very process contains little about innovation or invention, since it aims to modify and change some existing parameters of 3D scaffolds. This, however, is considered as nonideal when it comes to the design of novel scaffolds such as 3DPVSs. Therefore, a new methodology, which aims for innovation, will be of necessity, and it will be of higher value if traditional CAD methods used for traditional 3D scaffold design can be embedded within. 

After design initiation, concept generation and concept evaluation will come into play. We need to establish a proper methodology which could help generate the concepts of the 3DPVS as well as evaluating them. Ideal model of the methodology thus requires the ideal process of generating and evaluating the novel concepts. In other word, an ideal design process, as well as methodology, would highly accelerate the design innovation and help 3DPVS become more ideal from where it starts with. This is very likely a gradual process. In brief, an ideal design process including methodology can contain but not limited to following aspects. 

Firstly, the ideal methodology will fully cover all the stages for the conceptual design of 3DPVS, namely design initiation, concept generation, and concept evaluation. The conceptual solution of the 3DPVS after concept evaluation will be ideal, namely fully adequate, mature, and proper for the subsequent detailed design. Secondly, designers can use this methodology to fully fulfill different possible requirements, as well as solving possible challenges, in terms of the very conceptual design process, which means there is no need for designers to resort to strategies in other design processes or systems. Third, it requires the existing conceptual design strategies using CAD to be fully and adequately integrated into the new design process, which enables the new design process in two directions: to improve the parametric or functional properties of the traditional scaffold, for instance, the geometric, mechanical, and biological controls, as well as creating and triggering innovations and novel functionality following the laws of system evolution in order to help the scaffold reach a higher ideal level in its evolutional ladder. 

## 6. Conclusions and Future Work

This paper introduced the basic knowledge of the cell culture biomimetic scaffold, explaining its evolution and the positionality of the 3DPVS. The three-stage conceptual design process was introduced afterwards, and the authors put forward the scope of this paper’s work as focusing and studying on the design initiation, which can be of significant value at the current conceptual stage of 3DPVSs.

As the essence of this work, four aspects constituting the full stage of Design initiation have been studied. Firstly, the authors studied the fundamental aspects initiating the conceptual design of the 3DPVS, which includes the problem selection and requirement analysis, the fundamental relations of elements building up the conceptual 3DPVS, focusing on bone cells as the current application context and abstracting related cell culture requirements, as well as indicating the three-level system of the 3DPVS and the corresponding three-layer requirements for its design. Secondly, the authors introduced the GMBV characterization of the conceptual 3DPVS, which aimed to help analyze aspects of the 3DPVS in terms of its potential requirements, attributes, and components at different system levels. Vibratory properties would be consequently considered as the most unique feature of 3DPVSs compared with traditional scaffolds that remain passive or static. Thirdly, attempts were made to establish a potential scaffold base model of the conceptual design; three base models of the 3DPVS were selected, corresponding with the three different 3DP systems, namely the laser-, nozzle-, and droplet-based 3DP. Each base model has its unique benefits, which requires designers to analyze the specific case scenario before applying one or more base models as the starting point of design innovation. Fourthly, this paper discussed the ideality of the scaffold as well as establishing potential ideal models of the 3DPVS. The ideology of the ideal model corresponds to the knowledge of the base model. GMBV characterization was used to indicate the levels of ideality of the 3DPVS, and how to identify the objectives or ideal goals in scaffold design was summarized in a seven-step process. Such knowledge provides a clearer picture regarding the constitution of the ideal model, which could lead to better concept generation and innovation stages following design initiation. In this connection, the ideal model of the conceptual 3DPVS, regarding what properties it should have and what functionality it should ideally provide, was analyzed by this paper. Next to this, an ideal design process, with which the ideal model could possibly be achieved, was also discussed in brief.

In terms of future work, several directions could potentially be indicated. Following this design initiation study, future research is expected to have a solid foundation, where the work of 3DPVS concept generation and concept evaluation could start with. As mentioned earlier, concept generation would be the most innovative part inside the whole conceptual design process, which demands lot of energy, inspiration, and expertise from designers. Future researchers from different scientific or engineering realms, including bioengineering, vibration, 3DP manufacturing, mechanical engineering, and so on, are welcome to participate in the innovation work of 3DPVSs. Since nothing can be perfect, a further study on top of the current work of design initiation might be necessary in the future if new requirements, constraints, or possibilities in this stage of 3DPVS development can be put forward. 

## Figures and Tables

**Table 1:** Five perspectives focused upon by current design initiation.

Geometric Issues	Scaffold’s geometrics must cooperate with the new vibration mechanism to be designed, and the geometric control of the scaffold should stay at a predetermined state calculatable in both conditions when vibration is switched on and off. Unpredictable issues regarding geometric change during different working states of a 3DPVS could negatively alter the scaffold’s proposed functionality in a required cell culture scenario. As a result, geometric control of the 3DPVS and cell culture in static conditions could be similar to with a traditional 3D scaffold, while this would not be the case when the 3DPVS works in an active state.
Material Composition	Material composition of a 3DPVS very likely varies from that of traditional static or passive scaffolds. Materials of traditional scaffolds will be replaced partly or fully by active materials that help the scaffold generate desired vibrations. Analysis of material composition needs to be careful, as it not only ensures the scaffold has the required vibratory functions, but also maintains general functionality, such as proper mechanical strength and cell biocompatibility. A potential mistake made here, for instance, might be that the scaffold becomes sufficiently vibratory, while it is inapplicable in cell culture due to being poorly biocompatible.
Biological Requirements	In design initiation, biological requirements in terms of cell culture need to be fully and accurately understood. Compared with geometric or material properties, which could be separate traits of the scaffold, that is, both can be designed separately without altering the properties of another, biological properties of a scaffold tend to be the resultant property of both geometrics and materials. A thorough analysis of the required biological properties of scaffold in definite cell culture cases would help this issue.
Vibratory Dynamicity	The vibratory dynamicity proposed needs to be applied onto a 3D scaffold through an embedded, unseparated way, the approach to which might be different from traditional means involving connecting an external mechanical vibration onto the scaffold. A new way of thinking could therefore be necessary, and thus traditional tools for scaffold design, which chiefly focus on either geometric or material composition aspects, might not be inapplicable as they could not tackle the novel vibration issue as well as trigger the innovation which is the essence of a 3DPVS’s design.
3DP Fabricability	A 3DPVS needs to be fabricable through 3DP, which fulfills the essence of 3DPVSs as future products manufactured by novel 3DP methods. In this connection, a single 3DP method or a combination of different ones can potentially be utilized for fabricating the designed scaffold. Products being vibratory but not 3DP-fabricable will not be considered as having a proper design.
